# Obesity-related renal dysfunction: gender-specific influence of visceral adiposity and early impact of metabolic and bariatric surgery

**DOI:** 10.3389/fendo.2024.1440250

**Published:** 2024-10-14

**Authors:** Miruna Maria Popa, Anca Elena Sirbu, Elisabeta Andreea Malinici, Catalin Copaescu, Simona Fica

**Affiliations:** ^1^ Department of Endocrinology, Carol Davila University of Medicine and Pharmacy, Bucharest, Romania; ^2^ Department of Endocrinology and Diabetes, Elias Emergency University Hospital, Bucharest, Romania; ^3^ General Surgery Department, Ponderas Hospital, Bucharest, Romania

**Keywords:** obesity, renal dysfunction, metabolic and bariatric surgery, sleeve gastrectomy, visceral adiposity, body composition

## Abstract

**Introduction:**

Renal dysfunction is a recognized complication of obesity with an incompletely characterized pathophysiology. Improvement of glomerular filtration rate (GFR) after metabolic and bariatric surgery (MBS) has been reported across all classes of renal function. Inter-gender differences with regard to correlates of renal function have been described, but the influence of body composition is an understudied area. We aimed to explore determinants of renal function in obesity and to assess its variations after MBS, with a focus on body composition parameters in males and females, respectively.

**Materials, methods:**

We conducted a retrospective study on 196 patients who underwent laparoscopic sleeve gastrectomy, evaluated preoperatively and 6 months after the intervention. Recorded data included clinical and biochemical assessment, as well as body composition estimation via dual-energy X-ray absorptiometry. Serum creatinine-based formulas were used for the estimation of GFR.

**Results:**

We included a total of 196 patients (80 males and 116 females), with a mean age of 41.43 ± 10.79. Median baseline body mass index was 42.6 (6.61) kg/m^2^ and 6 months excess weight loss (EWL) reached 71.43 ± 17.18%, in females, estimated GFR correlated negatively with visceral adipose tissue (VAT) mass (rho=-.368) and this correlation was stronger in females with type 2 diabetes mellitus. Moreover, women in the third VAT mass tertile were 5 times more likely to have reduced GFR compared to the first tertile. Renal function improved after MBS across all classes of filtration. In males, this improvement correlated with EWL (rho=.358) and lean mass variation (rho=-.412), while in females it correlated with VAT mass variation (rho=-.266).

**Conclusions:**

Our results are consistent with previous findings on the positive impact of MBS on renal function and suggest a more prominent impact of visceral adiposity on GFR in females.

## 1 Introduction

Obesity is a multisystem disease, that has undoubtedly reached pandemic proportions. In 2020, there were over 800 million adults living with obesity worldwide and current trends predict a prevalence of over 1.5 billion in 2035 (1). Parallelling the obesity epidemic, chronic kidney disease is also a major cause of morbi-mortality worldwide (2). Multiple epidemiologic studies have shown that excessive body weight is a risk factor for both the onset and the progression of kidney disease across all ages and across a wide range of co-existing pathologies (3–9).

A more in-depth analysis of this relationship reveals that the two entities are, in fact, intertwined, involving complex and multilayered mechanisms of augmenting the detrimental effects of one another (3, 10–12). Thus, research in the field of obesity-related renal dysfunction is challenging and the issue remains incompletely explored, despite the fact that the interest in this topic dates from decades ago (13). The diversity of body composition among patients with similar body mass index (BMI), including varying proportions of adipose tissue components (e.g. visceral vs subcutaneous adiposity) is widely acknowledged (14). The distinct influences body compartments exert on renal parameters might prove useful in shedding more light on this adipo-renal relationship.

Metabolic and bariatric surgery (MBS) is currently the most effective intervention in severe obesity, with clear benefits on related comorbidities (15). Among these, the postoperative improvement of renal function has been reported in the past years and a number of authors regard MBS as a short and long-term renoprotective intervention (16). Specifically, MBS is associated with improved glomerular filtration rate (GFR), decreased albuminuria and a lower rate of progression to end-stage-renal disease upon follow-up (17, 18).

In the current study, we aimed to explore baseline determinants of renal function, focusing on body composition parameters, as well as short-term postoperative dynamics in patients with obesity undergoing laparoscopic sleeve gastrectomy (LSG).

## 2 Methods

We conducted a retrospective study on a cohort of adult patients with obesity who benefited from LSG at an International Federation for the Surgery of Obesity and Metabolic Disorders (IFSO) accredited Center of Excellence in Romania. The study followed the standards of the Helsinki Committee for Human Rights and the design received approval from the local ethics committee. All patients provided written informed consent. Evaluations were performed preoperatively and 6 months after the intervention.

Inclusion criteria consisted of adult age (>18 years old) and meeting the indication for MBS as per guideline recommendations (19). Exclusion criteria were represented by previous bariatric therapy, documented kidney disease, family history positive for hereditary kidney disease, usage of nephrotoxic medication during the past year and excessive alcohol consumption (defined as 5 or more drinks on any day or 15 or more per week for men and 4 or more on any day or 8 or more drinks per week for women) (20). Of the 268 patients initially included, 33 had incomplete baseline evaluation, while an additional 39 were either lost to follow-up or had incomplete evaluation during their second visit. Consequently, 196 patients were analyzed in the present study.

### 2.1 Clinical and biological evaluation

Body weight and height were measured (both at baseline and postoperatively) using a standardized digital scale with fixed stadiometer. BMI was calculated as weight (in kilograms) divided by the square of height (in meters). Excess weight loss percentage (%EWL) was calculated as [(Baseline BMI − Postoperative BMI)/(Baseline BMI − 25)] × 100, as defined in literature (21). Arterial blood pressure (ABP) was assessed in-office, at brachial artery level, with the patient in seating position, using an electronic sphygmomanometer. Hypertension (HTN) was defined as systolic ABP ≥140 mmHg, diastolic ABP ≥90 mmHg, or antihypertensive medication use following a previously established diagnosis. Postoperative improvement of ABP was defined as normalized ABP with consistent or de-escalated medical treatment. Diabetes was defined per American Diabetes Association criteria (22) or by the use of anti-hyperglycemic drugs or medical nutritional therapy following a previously established positive diagnosis. Improvement of glycemic control was defined as improved glycated hemoglobin by at least 0.5% and/or de-escalation of medical treatment.

Both pre- and postoperative assays were performed in the same laboratory. Creatinine was determined through colorimetric enzymatic assay and glomerular filtration rate was estimated (eGFR) using the CKD Epidemiology Collaboration (CKD-EPI) 2021 serum creatinine-based formula, adjusted for standard body surface area of 1.73 m^2^ (23). Classes of renal function were defined as: hyperfiltration (≥ 125), normal function (90-124), mild dysfunction (60-89), mild-to-moderate dysfunction (45-59 ml/min/1.73 m^2^), in accordance with KDIGO guidelines and commonly employed hyperfiltration cut-off values (24–26). Renal function was also estimated using the formula proposed by Basolo and colleagues (eGFR = 53 + 0.7 × (140 − Age) × Weight/(96xSCr) × (0.85 if female)), aimed at characterizing hyperfiltration (27).

### 2.2 Scan procedure—dual-energy x-ray absorptiometry

Whole body DXA scans using Lunar iDXA Forma (GE Healthcare) were performed to estimate total and regional body composition. Visceral adipose tissue (VAT) mass was quantified using the CoreScan™ application. Appendicular skeletal mass index (ASMI) was calculated as the sum of upper and lower extremities lean mass expressed in kilograms over the square of height expressed in meters. Quality assurance and control procedures were carried out in accordance with manufacturer indications. All scans were performed by an ISCD-certified DXA technologist, using the same software for analysis.

### 2.3 Statistical analysis

Statistical analysis was performed using IBM^®^SPSS^®^ v. 26.0. Parameter variations were defined as *value_6 months_ – value_baseline_*. Qualitative data was reported as frequency (percentage). Quantitative data was assessed with *Komologorov-Smirnov* test and normally distributed data was reported as mean ± standard deviation (SD), while parameters with non-normal distribution were described by median and inter-quartile range (IQR). Subsequently, independent and paired *t Student* tests were performed to compare normally distributed parameters, while *Mann-Whitney U* and *Wilcoxon Signed Rank* test were employed for non-normally distributed data. Bivariate correlations were explored using *Pearson’s r* and *Spearman’s rho*, respectively. Binary and linear adjusted regression models were employed to further explore the predictive value of parameters. Confounders were included as covariates in binary and multinominal regression, in order to validate the results. All analyses were 2-tailed, with a cut-off *p* value of less than 0.05 for statistical significance.

## 3 Results

The study included a total of 196 patients (80 males and 116 females), with ages ranging from 19 to 68 and a mean of 41.43 ± 10.79, slightly lower in males versus females (p=.08). The diagnosis of obesity had been established for an average of 18.21 ± 10.51 years, similar between the two genders. Median baseline BMI was 42.6 (6.61) kg/m^2^ and 6 months EWL reached 71.43 ± 17.18%, with no inter-gender differences.

T2DM and HTN baseline prevalences were 18.9% and 49.5%, with 6 months de-escalation of treatment reported in 51.3% and 43.2% of patients, respectively. Fasting plasma glucose, as well as total cholesterol, VLDL-cholesterol and triglycerides levels showed significant decline at 6 months.

Males tended to demonstrate greater 6 months percentual variations of body composition compartments - with the exception of total lean mass variation, which was similar between the two genders. **Table 1** summarizes the evolution of aforementioned parameters in males and females respectively.

**Table 1:** Pre- and postoperative values of selected parameters.

	Males, n=80			Females, n=116		
Parameter*	Preoperative	6 months	P value	Preoperative	6 months	P value
**BMI**	43.78 ± 4.84	30.61 ± 4.11	<.001	41.79 (6.44)	30.31 (4.63)	<.001
**Creatinine**	1 (0.2)	1 (0.2)	<.001	0.9 (0.2)	0.8 (0.1)	<.001
**eGFR (creatinine)**	93.94 (19.87)	102.48 (23.36)	<.001	83.15 (17.96)	89.59 (20.17)	<.001
**Total cholesterol (mg/dl)**	198 (66)	175 (42.74)	<.001	199.15 ± 37.79	192.32 ± 35.37	.03
**HDL-cholesterol (mg/dl)**	47.8 (13.5)	49.9 (11.3)	NS	56.47 ± 10.19	57.2 ± 10.34	NS
**LDL-cholesterol (mg/dl)**	106.7 (56)	107.4 (43.4)	NS	112.6 (38.7)	113.6 (42.9)	NS
**VLDL-cholesterol (mg/dl)**	32 (20.6)	20.6 (8.6)	<.001	24.5 (15.2)	17.9 (7.4)	<.001
**Triglycerides (mg/dl)**	163.5 (110)	104.5 (43)	<.001	122 (70)	89 (36)	<.001
**Fasting plasma glucose (mg/dl)**	93.5 (22)	79 (14)	<.001	94 (21)	79 (12)	<.001
**Total fat mass (kg)**	62.07 (15.71)	30.89 (11.81)	<.001	59.89 (14.11)	34.17 (9.89)	<.001
**Total lean mass (kg)**	71.82 ± 7.29	62.16 ± 6.71	<.001	52.22 ± 5.57	45.28 ± 5.09	<.001
**VAT mass (kg)**	4.4 (1.56)	1.58 (0.76)	<.001	2.38 (1.01)	1.06 (0.57)	<.001
**ASMI (kg/m^2^)**	10.49 ± 1.09	8.92 ± 0.89	<.001	8.79 ± 0.9	7.57 ± 0.77	<.001

* values are expressed either as mean ± SD, or as median (interquartile range), depending on the type of distribution.

BMI, body mass index, EWL, excess weight loss, eGFR, estimated glomerular filtration rate (determined by CKD-EPI 2021 creatinine-based formula); VAT, visceral adipose tissue; ASMI, appendicular skeletal muscle index.

Analysis of gender-specific determinants of renal function revealed a number of differences. Specifically, in females, eGFR correlated negatively with VAT mass (rho=-.368, p<.001), uric acid (rho=-.396, p<.001), glycemia (rho=-.419, p<.001) and VLDL-cholesterol (rho=-.239, p=.001). eGFR-VAT mass correlation was present irrespective of T2DM status, but was stronger in females with T2DM (rho=-.529, p=0.02). None of these results were replicable in males. Moreover, when dividing females according to menopause status (75% premenopausal, 25% postmenopausal), all of the correlations were lost in postmenopausal state.

To further explore the relationship between renal filtration and visceral adiposity, we compared renal function across VAT mass tertiles in females. Women in the third VAT mass tertile were 5 times more likely to have eGFR<90ml/min/1.73m^2^ compared to the first tertile (OR=5.2, 95%CI = [1.29,20.99], p=0.02), in a model adjusted for BMI, number of years living with obesity, ASMI, T2DM and smoking status. However, the model lost significance when also accounting for menopausal state. It should be noted that patients demonstrating hyperfiltration were excluded from this analysis in order to ensure adequate comparison.

**Figure 1:**
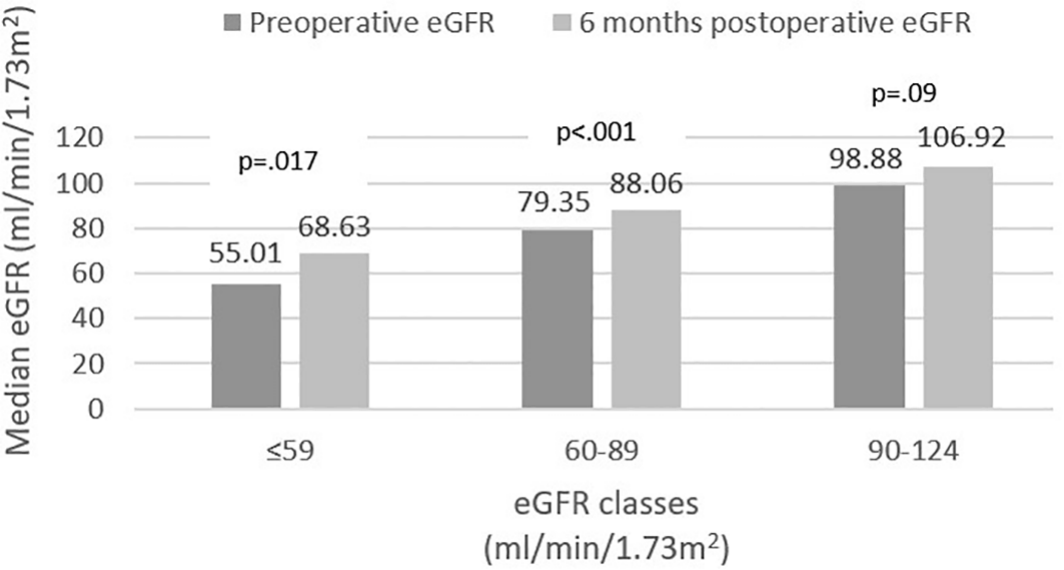
CKD-EPI (2021) estimated glomerular filtration rate (eGFR) variation.

**Figure 2:**
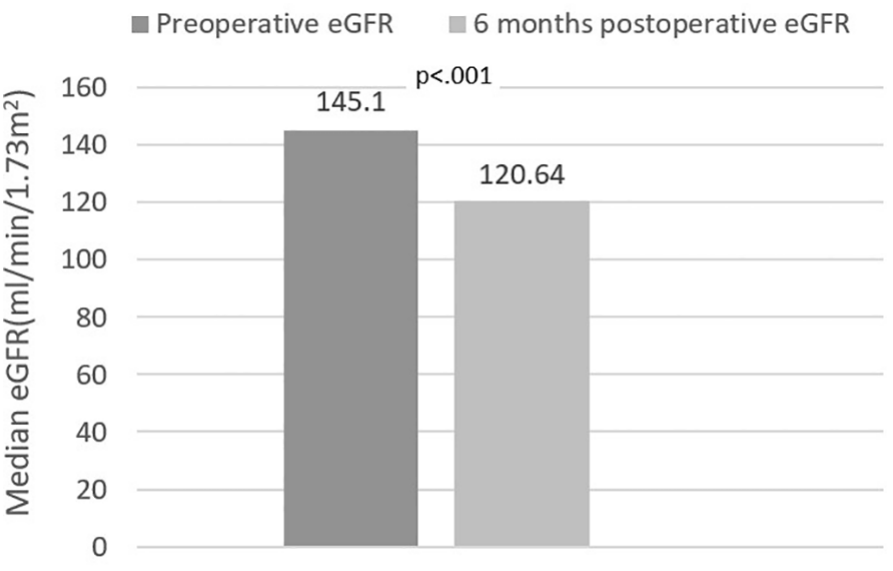
Estimated glomerular filtration rate (eGFR) variation in hyperfiltration class [eGFR formula - Basolo et al. (27)].

Stratification of baseline eGFR by standard classes of renal function revealed a very small prevalence of hyperfiltration in our group (1%), with the majority of patients demonstrating mild renal dysfunction (54.6%), followed by normal function (40.3%) and mild-to-moderate dysfunction (4.1%). Postoperatively, renal filtration improved across all defined classes, as depicted in **Figure 1**. Specific variations (expressed ml/min/1.73m^2^) were as follows: an increase from 55.01 (8.4) to 68.63 (20.97) in mild-to-moderate dysfunction class and from 79.35 (11.26) to 88.06 (19.64) in mild dysfunction class patients. In normal function class, baseline and 6 months eGFR values were 98.88 (13.75) and 106.92 (18.87), respectively (p=0.09). Finally, in the two cases exhibiting renal hyperfiltration per standard criteria, eGFR decreased postoperatively. The alternative approach of Basolo et al. (27) for evaluating hyperfiltration in patients with obesity determined the reclassification of 151 patients (77%) as exhibiting hyperfiltration and showed a significant postoperative reduction in their eGFR (145.1 (25.08) vs. 120.64 (28.55), p<.001) (**Figure 2**). We also considered a surrogate marker, eGFR/ASMI (aimed at controlling for postoperative muscle mass loss) and confirmed a similar pattern.

**Figure 3:**
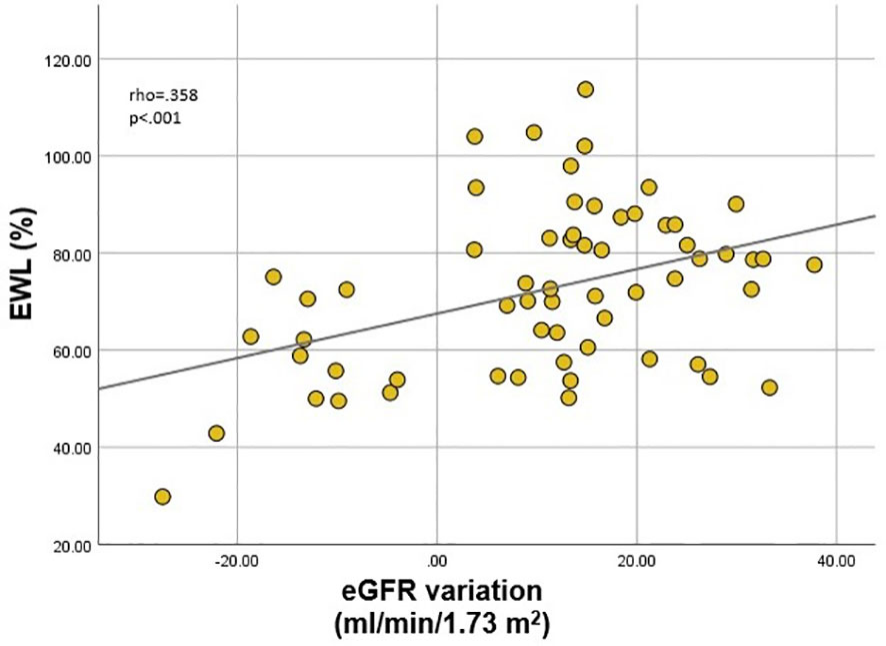
Correlation between estimated glomerular filtration rate (eGFR) variation and excess weight loss (EWL) in males.

**Figure 4:**
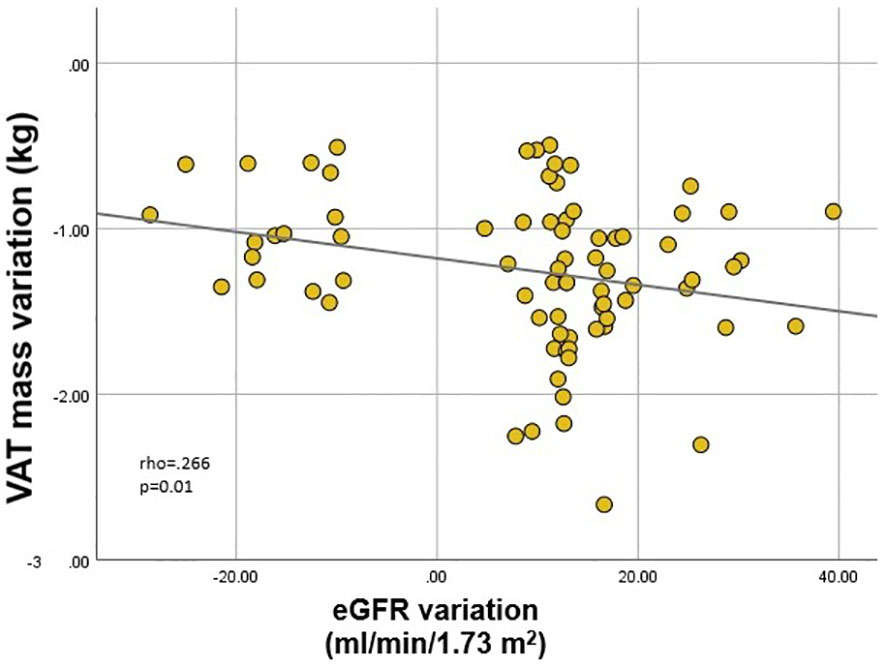
Correlation between estimated glomerular filtration rate (eGFR) variation and visceral adipose tissue (VAT) mass variation in females.

Finally, we analyzed correlates of renal function variation in all patients exhibiting either increase or decrease of eGFR. Inter-gender differences were evident once more. In males, eGFR variation correlated (p<.001 for all) with EWL (rho=.358) (**Figure 3**), lean mass (rho=-.412) and ASMI (rho=-.374) variations; in females, on the other hand, eGFR variation only correlated (p=0.01) with VAT mass (rho=-.266) (**Figure 4**) and LDL (rho=-.279) variations. VAT mass variation was an independent predictor (β=-.246, p=.02) of eGFR variation in females in a model adjusted for baseline eGFR, menopause status, EWL, ASMI variation, LDL variation and additionally for the improvement of both T2DM and HTN (model data: R=.6, R^2^=.36, p<.001). Conversely, in males, EWL was an independent predictor of eGFR variation (β=.32, p=.01) in an equivalent model – adjusted for baseline eGFR, ASMI variation, VAT mass variation and for the improvement of T2DM and HTN (model data: R=.63, R^2^=.4, p<.001).

## 4 Discussion

In the present study we investigated the relationship between creatinine-based estimated glomerular filtration rate and DXA-evaluated body composition, as well as basic biochemical parameters in a cohort of patients with severe obesity undergoing LSG. Gender-oriented analysis identified interesting differences in the factors influencing renal function in males vs. females, both at baseline, as well as when considering early (6 months) postoperative variation.

Estimation of renal function by means of serum creatinine levels is currently the most widespread method used in clinical practice for both screening and monitoring and it also constitutes the basis of renal function staging (28). The CKD-EPI formula is believed to yield more accurate results and is, hence, favored by profile societies (23, 24, 28, 29). Its main limitations derive from the influence of muscle mass and dietary protein intake on serum creatinine levels (30), from the delayed increase in creatinine compared to the onset of renal function decline (31) and from the fact that the equations were developed on a population mainly in the normal BMI range (32). These lead to inaccuracies both in the general population with obesity, as well as in assessing the dynamics after MBS (31–35). Various means of resolving these shortcomings have been proposed in literature, with mostly unsatisfying results. The most popular is de-adjusting from standard body surface area (BSA) of 1.73m^2^ and adjusting for calculated BSA (de-indexed equations). However, this method consistently overestimates renal function, since muscle mass, and not total body mass is actually the main contributor to the estimation bias (36, 37). In this regard, Nankivell and colleagues demonstrated that ASMI constitutes the best correlate of eGFR error (34). Considering these results, we used the eGFR provided by CKD-EPI (2021) formula, adjusted for standard body surface area and corrected our analyses for ASMI and ASMI variation in order to provide accuracy. Through this, we were able to stratify patients in classes of renal function and assess their dynamics in compliance with guideline recommendations and in line with standard clinical practice. Renal dysfunction is reported to be more prevalent among persons with obesity (11, 38, 39) and our results are in line with previous findings, with the majority of patients exhibiting mild or mild-to-moderate dysfunction at baseline. An ample study, with an 8 million person-year follow-up, elegantly demonstrated that the risk of end-stage CKD progressively increases with the increase of BMI. Particularly, patients with severe obesity had a 7-fold increase in risk compared to normal weight controls (4). Additionally, in a more recent meta-analysis comprising a total of 3 million patients, obesity was associated with an almost two fold increase in the risk of renal function decline) over a 10 year follow-up (6). The largest epidemiologic study regarding CKD in the general Romanian population reported a cumulative prevalence of reduced kidney function (defined as eGFR<60ml/min/1.73m^2^) of 3.76% (40). Comparatively, in our cohort, mild-to-moderate renal dysfunction had a prevalence of 4.1%. However, the majority of our patients (56.4%) exhibited mild dysfunction, on which the aforementioned study did not report.

Postoperatively, we identified significant increase in eGFR in patients exhibiting subnormal renal function at baseline; this was paralleled by a statistically insignificant (p=.09) less ample eGFR increase in patients classified as having normal baseline renal function (**Figure 1**). Interestingly, the patients exhibiting hyperfiltration, demonstrated postoperative decrease of eGFR; however, the very small number of subjects pertaining to this category precludes these results from being statistically relevant. Based on the assumption that currently used formulas underestimate the prevalence of renal hyperfiltration (36, 41), Basolo and colleagues devised a new formula, specifically aimed at more accurately identifying hyperfiltration in patients with obesity (27). Using this approach, we reclassified the majority of the patients (77%) as exhibiting hyperfiltration and confirmed a significant postoperative decrease of eGFR in this class (**Figure 2**). However, the authors specifically excluded patients with renal dysfunction from their original analysis; therefore, while these results provide further confirmation of eGFR improvement across all renal function classes, re-thinking the stratification of all patients based on the proposed formula is likely to be inaccurate. These outcomes were also confirmed by the comparable variation of eGFR/ASMI [analogous to the procedure employed by Favre and colleagues who used eGFR over lean body mass in their study (42)]. There is currently a robust body of literature with similar results, pointing to the beneficial role of MBS-induced weight loss on renal function (16–18, 33, 43–45). Improvement of GFR and proteinuria are evident in the first months-to-years following surgery, but positive effects have been recorded up to 15 years postoperatively (43).

Obesity negatively impacts renal dynamics through a combination of direct and indirect mechanism; of the latter, the two most important are HTN and T2DM, both of which are widely recognized complications of obesity and also major risk factors for the development of kidney disease. However, direct pathogenic ways, occurring even in the absence of overt HTN/T2DM are believed to be of equal importance. These include hemodynamic alterations and increased sodium reabsorption (leading to initial hyperfiltration, followed by GFR decline), adipokine and cytokine dysregulation, insulin resistance, oxidative stress and direct lipotoxicity (3, 10–12). Visceral adipose tissue (VAT) is recognized as the metabolically active, pathogenic compartment, linked to aforementioned alterations (46), as opposed to subcutaneous adipose tissue (SAT), which serves as a buffer for excess energy and has positive metabolic impact (47, 48). Obesity-related adipose tissue dysfunction is characterized by chronic inflammation and M1-polarized macrophage infiltration and activation, all of which occur predominantly in VAT (47, 49). Kidney morphology and physiology (e.g. high vascularization, not paralleled by equally effective local anti-inflammatory guards) determine increased renal sensitivity towards inflammation-induced damage (50). Studies have tied various pro-inflammatory molecules (e.g. tumor necrosis factor-alpha (TNF-α), interleukins 1β and 6 (IL-1β, IL-6) etc.) to the onset of chronic kidney disease (CKD) (50). A landmark study including over 3000 patients revealed that higher circulating TNF-α and fibrinogen levels are associated with more rapid decline of eGFR in patients with established CKD (51). More recently, involvement of TNF-α in renal fibrosis, as well as the benefits of its inhibition have been described in murine aristolochic acid-induced nephropathy (considered superior to previously employed murine models) (52). Moriconi et al. aimed to assess the relationship between IL-1β/Caspase-1, insulin sensitivity and early-stage obesity-related renal damage, namely hyperfiltration. Apart from demonstrating the capacity of IL-1β to predict hyperfiltration in patients with severe obesity, their study had another valuable result – patients in whom GFR did not normalize after MBS also showed failure to normalize circulating IL-1β/Caspase-1 levels (53). These results suggest a pathogenic role of the inflammasome in kidney dysfunction, as well as the potential for achieving renoprotection through targeted anti-inflammatory therapy, in concert with weight reduction. Similar strategies are being considered in diabetic kidney disease, in which renal benefits of medication with potential anti-inflammatory effect (e.g. type 2 sodium-glucose cotransporter inhibitors, glucagon-like peptide-1 receptor agonists, renin-angiotensin-aldosterone system (RAAS) inhibitors) are evidently superior to those of medication that solely achieves tight glycemic control or reduction of arterial blood pressure (54).

Apart from the mechanisms detailed above, it is noteworthy that peri- and intrarenal accumulation of VAT also increases local pressure, activating sympathetic and RAA systems and directly contributes to glomerular lesions (55, 56). In line with these concepts, central adiposity (i.e. various estimates of VAT), perirenal adipose tissue and renal sinus fat exhibit a stronger correlation with the occurrence of obesity-related renal complications than BMI or subcutaneous adipose tissue (57). In a multicentric study using bioelectrical impedance analysis aimed to assess the ability of various obesity indicators to predict renal impairment (GFR decline/new-onset proteinuria) during a 6-year follow-up, only visceral fat area (with a cut-off of 100 cm^2^) proved to be a sensitive marker (58). Kataoka and colleagues found that VAT to SAT ratio serves as a predictor of eGFR decline in CKD patients, with a greater impact in females and in patients with lower absolute values of VAT (59). Interestingly, the relationship between VAT mass and CKD development seems to be stronger in patients with lower body weight. In a large scale cohort study (11000 subjects, 5.6-year follow-up), VAT was a predictor of CKD onset only in patients with normal BMI (60).

Accurate estimation of VAT mass has been a topic of great interest in the past years and surrogate markers such as lipid accumulation product (LAP), visceral adiposity index (VAI) (61), novel VAI (NVAI) (62), Chinese-population specific cVAI (63) and, more recently, the metabolic score for visceral fat (METS-VF) (64) have been widely used in studies of obesity-related renal affliction. However, DXA has the advantage of providing a direct and accurate assessment of body composition and the CoreScan™ application has been validated against MRI and CT, with satisfactory concordance (65).

In a meta-analysis including seven studies, Fang and colleagues concluded that VAI is a useful tool for predicting CKD (area under the curve 0.77, prediction rate of 73% when considering a 50% pre-test probability) (66). Another ample study revealed that both LAP and VAI are associated with increase odds of CKD incidence (61). Interestingly, in our study, the correlation between eGFR and VAT mass, along with various other markers of lipotoxicity and inflammation (uric acid, VLDL-cholesterol) was only apparent in females, and not in males. Moreover, when analyzing VAT mass tertiles, the odds of reduced eGFR (<90ml/min/1.73m^2^) was 5 times higher in women in the third VAT mass tertile compared to those in the first one, in a model adjusted for major confounders. This is in line with other reported findings. For example, in an ample community-based study in Taiwan, involving approximately 2000 subjects, VAI was significantly associated with the presence of CKD in females, but not in males (67). In another large study, comprising 5355 subjects, Xu and colleagues demonstrated a negative correlation between eGFR and CVAI and found CVAI to more accurately predict renal dysfunction in females compared to males (63). Similarly, in a study involving 400 middle-aged and elderly subjects, VAI was an independent predictor of CKD in females, but not in males (68). Another interesting study based on the KORA cohort, revealed a negative association between cystatin-based eGFR and MRI estimated VAT only in women. However, the authors found no correlation between creatinine-based eGFR and VAT (69). Similarly, in a study including approximately 35000 participants from the NHANES database, no association between VAI and eGFR could be established; nevertheless, visceral adiposity was associated with increased odds of chronic kidney disease and albuminuria in females (70).

In contrast, a number of studies reported negative correlation between VAT and renal function in males, rather than in females. For example, in a longitudinal study including almost 7000 non-diabetic patients, VAI was found to be an independent predictor of renal function decline only in men over a period of follow-up of 8.6 years (71). Similarly, in a cross-sectional study conducted by Seong et al. and including almost 5000 subjects, VAI and LAP correlated with CKD exclusively in males (72). Yu and colleagues found METS-VF to be an independent predictor of CKD, exhibiting superiority to other obesity indices in both sexes, but more prominently in men (73).

Males and females differ not only in body size, but also in body composition. Men tend to have higher BMIs, as well as a higher absolute value of VAT and VAT: SAT ratio when compared to premenopausal women (74, 75). Similarly, male and female kidneys show differences that go beyond size, namely in histology and physiology (76, 77). For example, inter-gender variability in the abundance and functionality of renal transporters has been described (77), explaining differences in water and solute handling (e.g. a more rapid and efficient natriuretic response to high salt diet in females (78)). These provide a basis for the discrepancies in CKD evolution (known to be slower in premenopausal women than in men (77)) on one hand, and might explain the gender-specific renal-VAT interaction, on the other. Gonadal hormones are likely to have the greatest impact on these dissimilarities, influencing both body composition parameters and renal function directly. Estrogen promotes expansion of SAT, rather than VAT, reduces RAAS activation, promotes nitric oxide- mediated vasodilation and attenuates inflammation (75, 79). Of note, three types of renal estrogen receptors have been described and estrogen is believed to also exert its direct renoprotective actions, of which we mention attenuation of glomerular hypertrophy, reduction of mesangial expansion and decrease of fibrosis, mainly via ERα (80–83).

Menopause brings about a reversal of the female “advantage” in adiposity polarization and CKD progression, further supporting the central protective role of estrogen (81). The global consequences of estrogen decline (e.g. inflammatory milieu alterations (84)) further enhance the negative renal impact. In line with these findings, in our study, menopause status influenced all preoperative correlates of renal function. An ample prospective study, with a 15-year follow-up, interestingly showed that women with low estimated endogenous estrogen exposure had a higher risk of developing CKD later in life (79), while, in a nationwide Korean study, the use of menopausal hormone therapy was associated with a reduced risk of end-stage renal disease (83). However, in the context of obesity, conflicting results have been reported, with a number of deleterious effects especially in female subjects (85–87). Moreover, the dysregulation of systems involved in estrogen-mediated renal protection (e.g. RAAS) (88) might render estrogen less effective in the context of obesity (75). In their studies on murine models, Rodríguez-Rodríguez and colleagues elegantly assessed the combined renal effects of obesity and menopause. Ovariectomized obese mice had the most detrimental metabolic and renal responses to obesogenic diet and also exhibited the most severe lipotoxic and inflammatory profile in renal tissue (81, 91). These results suggest concerted deleterious renal effects of obesity and menopause and highlight the importance of weight control in menopause and perimenopausal transition as a renoprotective measure.

Another noteworthy result of our research is the stronger eGFR-VAT mass correlation in female patients with obesity and T2DM versus female patients with obesity, but no T2DM diagnosis. A very recent study by Liu and colleagues using DXA scans to quantify visceral adiposity in patients with T2DM, revealed additive interaction between diabetes and visceral adiposity on the occurrence of albuminuria. Higher visceral adiposity was also found to induce a stronger correlation between T2DM and albuminuria (89).

There are multiple hypotheses regarding post-MBS renal function improvement, derived from the positive impact on all aforementioned obesity-related dysregulations. Most of the studies found that improvement of renal filtration does not correlate with the degree of weight loss, but rather with the amelioration of metabolic profile and comorbidities, as a consequence of VAT and ectopic adipose tissue reduction (35, 43, 44, 90). However, we revealed a positive correlation between eGFR variation and EWL in male patients with obesity, paralleled by negative correlations with lean mass and ASMI reductions (which may be considered logical in light of the influence on creatinine). These results were not replicated in females. Instead, similar to the baseline strong influence of visceral adiposity, VAT mass variation proved to be an independent predictor of eGFR variation in a model adjusted for the main confounders. Interestingly, this held significance regardless of menopause status, suggesting beneficial effects of VAT mass reduction in both states.

To our knowledge, no other studies have reported on the correlation between DXA-estimated VAT and GFR dynamics before and after MBS, neither globally, nor through gender-oriented analysis. The availability of DXA also provides an additional strength of our study, namely the possibility to assess and adjust for the influence of ASMI. The main limitations stem from the inherent issues with creatinine-based eGFR, as discussed above. Although ideal, direct measurement or GFR is unfeasible in clinical practice. However, estimating GFR via equations based on both creatinine and cystatin C might increase reliability (31, 32). Secondly, albuminuria was not assessed in our cohort and therefore we could solely analyze renal function, rather than chronic kidney disease. Finally, while benefiting from a fairly ample evaluation, there are still confounders that should be considered when interpreting these results, such as dietary intake, physical activity levels, and socioeconomic status, which may influence both obesity (along with body composition parameters) and renal function (including the estimation of GFR).

The present study concentrated on the early impact of MBS on renal function. Added value would be provided by long-term assessment. However, the most significant improvement in GFR seems to occur in the early postoperative timeframe (6-12 months), as pointed out by a meta-analysis by Huang et al. (17). Finally, our results should be confirmed on larger and more diverse cohorts, since we included a fairly low number of patients pertaining to the same ethnic group.

## 5 Conclusion

In the current study, we demonstrated a positive impact of MBS on obesity-related renal dysfunction and outlined gender-specific determinants of baseline eGFR and of postoperative eGFR variation. Our results suggest a stronger impact of visceral adiposity and lipotoxicity on renal pathology in females with obesity. This warrants further exploration, as it may guide future personalized therapeutic approaches.

## Data Availability

The raw data supporting the conclusions of this article will be made available by the authors, without undue reservation.
